# Selective genetic targeting of the mouse efferent vestibular nucleus identifies monosynaptic inputs and indicates function as multimodal integrator

**DOI:** 10.1152/jn.00467.2025

**Published:** 2026-03-02

**Authors:** Miranda A. Mathews, Victoria W.K. Tung, Emily C. Reader-Harris, Andrew J. Murray

**Affiliations:** 1 Sainsbury Wellcome Centre for Neural Circuits and Behaviour, University College London, London, W1T 4JG, UK; 2 School of Biomedical Sciences, Faculty of Biological Sciences, University of Leeds, Leeds, LS2 9JT, UK

**Keywords:** Efferent vestibular nucleus, monosynaptic rabies tracing, viral targeting, sensory systems, CGRP

## Abstract

The vestibular system is a critical sensory modality required for coordinated movement, balance and our ability to interact with the surrounding environment. Vestibular sensory neurons provide the nervous system with information about head rotation and acceleration. However, the nervous system can also modify the activity of sensory neurons and hair cells via the actions of the efferent vestibular system (EVS). The function of the EVS has remained unknown partly because of an inability to target efferent vestibular neurons in a selective manner to understand their synaptic inputs and function during behaviour. Here, we present a novel method for the selective targeting and expression of flp-recombinase in EVS neurons. We take advantage of the dual expression of choline acetyl transferase (ChAT) and calcitonin gene related peptide (CGRP) in these neurons to develop an adeno-associated virus (AAV) that expresses a gene only in neurons with this intersectional expression. We use this system to map the monosynaptic inputs to EVS neurons and show inputs from distinct populations of brainstem and midbrain regions indicating a functional role as a multimodal processing center and integrator for the vestibular periphery. To demonstrate the applicability of our technology in behavioural assays, we performed a preliminary behaviour analysis (treadmill running and open field) in mice with disrupted EVS function. While more bespoke assays are required to ascertain EVS function/s, our viral method presents a novel tool for investigators examining the role of the vestibular system and its central circuits.

## Graphical abstract

**Figure:**
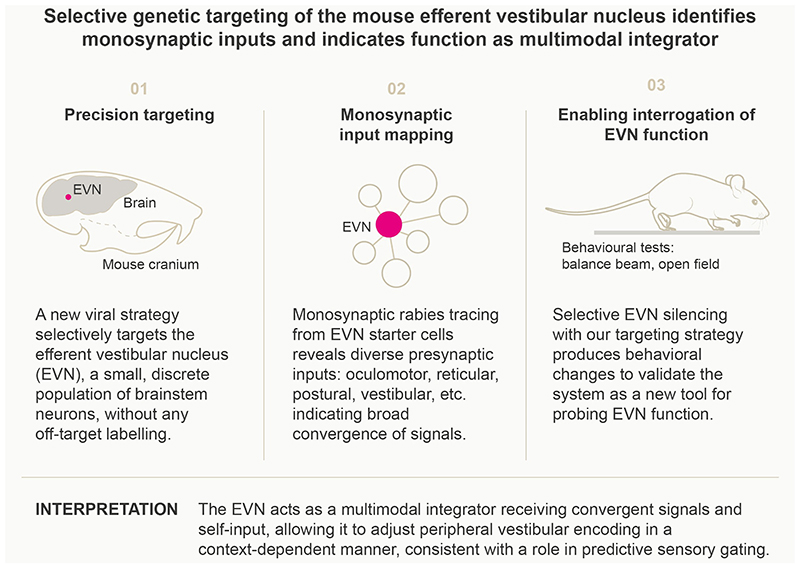

## Introduction

The vestibular system is indispensable for the coordination of movement, balance and our general ability to interact with the environment. This ‘sixth sense’ provides information regarding head rotation and acceleration, which is combined in the brain with other sensory modalities to give a sense of the position and movement of the body in space.

Anatomically, vestibular organs for all land vertebrates are in the inner ear and vestibular sensory neurons relay information to the brainstem. Aquatic vertebrates possess an additional system, the lateral line, that detects changes to the surrounding aquatic environment (such as pressure, vibration, movement) and is critical for orientation underwater. Like several sensory systems communication is bidirectional, where the central nervous system can exert influence over the peripheral sensory organs. A central modulation of vestibular end organs has been observed across all vertebrates – and is termed the efferent vestibular system (EVS). The EVS originates in the efferent vestibular nucleus (EVN) of the brainstem and EVN neurons terminate directly on vestibular hair cells and afferent sensory neurons. In amphibians, efferent fibres innervate both the inner ear and lateral line (Hellmann and Fritzsch, 1996). However, while we know the EVS has specific cellular actions on vestibular and lateral line sensory receptors (for reviews, see Mathews et al., 2017; Holt et al., 2010), a specific behavioural and functional role is yet to be understood.

Over the last seven decades, researchers have adapted a variety of *in vivo* and *in vitro* techniques to understand the function of this small and evolutionarily conserved nucleus. These experiments fall into three broad categories: 1) EVS stimulation (e.g. chemically, electrically or thermally (most recently in Raghu et al., 2019) combined with recording peripheral vestibular activity (summarised in Mathews et al., 2017); 2) putative physiological EVN recordings under different behavioural paradigms (some examples include, Russell, 1971; Highstein and Baker, 1985; Plotnik et al., 2002; 2005; Chagnaud et al., 2015); and 3) mouse transgenic lines combined with behavioural recordings (Luebke et al., 2014; Hübner et al., 2015; 2017; Chang et al., 2021). Collectively, these works give insights into the potential function of the EVS (for reviews, see Holt et al., 2010; Mathews et al., 2017; Mathews et al., 2020; Poppi et al., 2020; Cullen and Wei, 2021), especially when paired with direct physiological recordings of EVN neurons (Leijon and Magnusson, 2014; Mathews et al., 2015). However, a complete understanding of EVS function requires an ability to target and manipulate EVN neurons in awake behaving animals – which so far has not been possible.

Recombinase mouse lines, where cre or flp is expressed in genetically defined neurons, have revolutionised our understanding of behavioural roles of neuronal subtypes. In the EVN, which is a cholinergic nucleus, Lorincz et al., 2021 utilized a ChAT-Cre mouse line to perform anatomical tracing of EVS neurons. However, cholinergic neurons are widespread in the brain and perform multiple functions so behavioural assessment of EVS function requires a finer scale of neuronal subtype resolution. Similarly, short enhancer or promoter sequences can be used within viral vectors such as adeno-associated virus (AAV) to drive cell-type-specific gene expression. In addition to ChAT, calcitonin gene related peptide (CGRP) is known to be expressed in EVN neurons (Mathews et al., 2015). Several groups have described CGRP promoter fragments for use in sensory or central neurons (Watson et al., 1995; Messina et al., 2000; Durham et al., 2004; Shimizu et al., 2017), providing an opportunity to utilise these same sequences for EVN targeting.

Here, we combine the ChAT-Cre transgenic mouse line with both cre-dependent AAVs and a cell-type-specific short promoter to intersectionally target brainstem neurons expressing both ChAT and calcitonin gene related peptide (CGRP). This intersection permits selective targeting of EVN neurons, which co-express both ChAT and CGRP (Ohno et al., 1991; Mathews et al., 2015). We first validated this approach by retrograde labelling of EVN neurons by applying fluorogold to the end organs. Following this validation, we were able to use this novel approach to identify the monosynaptic inputs to EVN neurons using modified rabies virus (RABV) and separately block neurotransmitter release from the EVN through the directed expression of tetanus toxin light chain (TeLC). This approach demonstrates that the EVN can be selectively targeted, providing a novel tool to further explore role of the EVS in gross motor and vestibular coordination.

## Materials and Methods

### Generation of AAV constructs

AAV constructs using the CGRP short promoter were based on a promoter sequence from Durham et al. (2004) and Thomas et al. (2001). We de novo synthesised (GeneArt, Life Technologies) a 2Kb region immediately upstream from the CGRP-β coding region. The sequence of the promoter region is as follows:

5’ ACGCGTGGTACCCCTGCCAGCTGTCGGATGCCTGAACCTATGGATGCTTAACCAGCAGCTGAGTGAGTGTGA AAGTTCCCTGCTGACCCCTGGGTTGCTCAGGTGGCCTGTGCAGGACCCTGGCTCAGCTCTCAAGCTCTGCAGA GTGGGGTGGTGGAGATGCAGGGGAGGGGAAGGGAACTGCCATCTGAGCGCCAGCCTCCTGCTAGGCAAACC CGCCAGGGATGCTTGGAAGTGCTTTAATCTACACTGCTACAGCAGTGTGAGGTCTGGGGATTTGAATGGGGG CGGGGGAGGGATGTAAAAACCATAGCGCAGGATTTGGAAGGTCTGGTACAGGAGGAGAAAGCCCAGTTCCC TGTGCAGTCTGTTAGCCTGCTGCTCCACAATACTCTCTAGTTTCTATCCTTATTAGGTGATGGGAAGCACGCAC TGCTAGAGTGCCCATTTGGGACAGGTATGACAGAAGTACCCTAATGTATCCAAGGACCCGCTTCTTCCTGTGA CAGTCATCATCGTGGATGTATCTACTGAAGTCCTTTTAGAACCTGGGAGTGCTACTCAGCCTGCGTGGGAGTC CAGCTACGAGGTTCAGGTCCCCATTGGAGTGGGCAGCAAAAGGTTGTAGGCTGGAGTTCAGGTATTAAAGAG GTCGTGATGTCAAACTCAGGTTTGCTCACATTCTGGACGAATTCACCCTCTCTGTATCCTTACCCCACCCCCACT CCCACTCCTACCCGGTTCCTCAGCAATGACCTCAAAGACAGGGAGTGGACTGCTGCCTCCCTCCTGCAGAAGT GTAAGTAGCTCCAGCTATGACGTTATGGAAGCTCGGTCAGAGCTCTGATTGGTGGAAGAGCTACTGCGGACC CCCCACACCCTCAAGATCGAGAATAAGAGACCACGGCTCTGGGGACAAGACGCCTACAGCCGTGTGTGTGTG CTCTTCTGCAGTGGACACTTCACTCTCGCTGTTCCAACACGGGCTAGCAGGTGAGAAACTTAACTTCTCAACGC CTACAGCTCTCTCTCCTTTAGTTTGTTTCCTTTTGGTTGCTCTTTTTAATGCAGTATTTCACACTGTAGTCTAAGTT GGCCTGGCACCCACTATATAGCTCAAGTTGGTTCGTCTCAAACGCTAAGGTTCTCCTTTCTCAGCATCCTAAGG ATGCGGATTACAGGCGCAAGCCACCACACCCCACTCTACTTGGATCCCTTTGCTGTCCTGGTTCCTTATCATTCC ACATACATTTCCGCCTTCCTGCAGCCATTGTCAGAAAGTACAGTCTTGACATTTTCTTTTAACTAAAGTAAGTGG GACCCCTACGACTACTCAGCAGCACTGGAAGCTGGGCGACCCTATCTAGGCGCGTCTGTGCCCCCTCCTTGAG GGAAGGTGGTCTTGCCGCATCCTAACCAGTTTTAGGTTAAAGAGTTCCTTGGATTCGGGATTGGGGAGCACTG ATCTTTTCTCTCAGATGTTTCCAGCCTTTAGCCTCCTGGGGTTATCAGCAAGCAGGTGGGTCTCGCTTCGCTGT GGGGAGGAGGAGTCCTCATCTGCGGTTCTGAGGTAGTTTAAAAAAAAAAATCTCCCAACTCTGCAGATGGAG AGAGGGGGATTAGTTCCAAGTTAACTTTCTTCCCCAGGGCAACCTCTCAGAAAGGGTGATTATAATAATTTCA ACCTGTTAGAAATCCTTAGCAGCGGGACAGCAAGGCGCAGGGATCTCTGGGTGGTTTTTGGTTTCTTCACAGA TGAGAGCCAAAGGGGCGCGGCACGTGTGTTCTCCTGCAAGCTGGGGGCAAATGAGTGCCGGTAGCTCCTCCT TGTTCTTAAACCGAGCAGAAACTGCAAACCACATCTACTCTCCCCCACTCGTTTCTGCTCTATCAAGCCACTCAC CACACTGCATCTACTGCACGTTTTGAGAGCTGCAGTGTGGTAGGAGAAATAGAACCTGGGTCTATAGTCCTGA GCAATTGGACCATTCTTCTCTTCTTACAGAGACATCTTAATTAACTAGTGCGGCCGCCACCTCTAGAGGATCC 3’

The vector pAM-CGRP-FLPo-HA was used for initial testing of the CGRP promoter. DNA was de-novo synthesised containing the entire 2Kb CGRP promoter region upstream from FLPo, a 2A self-cleaving peptide sequence and 3x HA tags. This entire sequence was cloned into a vector containing AAV2 inverted terminal repeats (ITRs) (Murray et al., 2011) via KpnI and HindIII restriction sites.

The CGRP and cre-conditional FLPo or GFP (pAM-CGRP-Flex-FLPo or pAM-CGRP-Flex-GFP) were generated by replacing the CAGGS promoter in pAM-Flex-Empty (a cre-conditional vector containing AAV2 ITRs; Murray et al., 2011) with the CGRP short promoter via KpnI and XbaI restriction sites. FLPo or GFP was cloned in reverse orientation into the multiple cloning site using EcoRI and XhoI restriction sites.

### Generation of recombinant AAVs

AAV vectors used in this study were all packaged according to McClure et al., 2011 with some minor modifications. Briefly, human embryonic kidney (HEK) 293 cells were transfected via either calcium phosphate or Turbofect (Thermo Fisher Scientific) with individual AAV backbone plasmids as well as a 1:1 ratio of AAV1 (pH21) and AAV2 (pRV1) or AAVDJ helper capsid proteins and adenovirus helper plasmid pFdelta6. 48 hours post-transfection, the cells were harvested and AAVs purified using 1mL HiTrap heparin columns (Sigma), concentrated using Amicon Ultra centrifugal filter devices (Millipore) and purified using an AAVpro Purification Kit (Takara Bio).

The recombinant AAVs we designed and produced for selective EVN targeting were, in order of experimental use:

1) AAV[2/1]-CGRP-FLPo-HA
2) AAV[DJ]-CGRP-FLEx-GFP
3) AAV[DJ]-CGRP-FLEx-FLPo

Other AAV vectors used in this study were:

- AAV[DJ]-Ef1a-frt-H2BG-TVA (for rabies monosynaptic tracing; Reardon et al., 2016)
- AAV[DJ]-Syn-frt- H2BG-N2cG (for rabies monosynaptic tracing; Reardon et al., 2016)
- AAV[DJ]-EF1a-fDIO-TeLC-GFP (for behavioural recordings after blocking EVN transmission; ETH Zurich Vector Core)
- AAV[DJ]-EF1a-fDIO-GCaMP6s (control for behavioural recordings with TeLC injections; Addgene plasmid #105714)

### Animals

All animal experiments were performed under UK Home Office license (PPL: PE4FA53CB) according to the United Kingdom Animals (Scientific Procedures) Act 1986. Male and female mice aged between 8-16 weeks old from C57BL/6J and B6J.ChAT-IRES-Cre::Δneo (ChAT-Cre; JAX stock no.: 031661; Rossi et al., 2011) homozygous animals were used for experiments.

Initial experiments used ChAT-cre::tdTomato mice that were generated in house from a cross between homozygous strains B6J.ChAT-IRES-Cre::Δneo and ROSA-loxP-STOP-loxP-tdTomato (Madisen et al., 2010). These mice expressed tdTomato exclusively in cholinergic neurons and were used to demonstrate the location of EVN neurons (ChAT-positive) with peripheral fluorogold labelling.

All animals were housed in temperature-controlled environment with a 12hr light/dark cycle (red lights on at 0900) and free access to food and water. Surgeries and behavioural experiments were carried out during the dark phase of the cycle. All efforts were made to minimise animal suffering.

### Surgical procedures

#### Stereotaxic injections

Stereotaxic injections into the EVN were made as described previously (Murray et al., 2011), adapted from Cetin et al. (2006). Briefly, mice were anesthetised with isoflurane in oxygen (4% induction; 2% maintenance during surgery) and given a subcutaneous injection of analgesics (Metacam at 5mg/kg). The mouse was fixed in a stereotaxic frame (Model 942, Kopf, USA) and an incision was made in the skin such that bregma and lambda could be exposed and visualised. A small burr hole was made above the injection site (relative to bregma) where viruses were injected using a pulled 3.5” borosilicate glass capillary with 8μm bore width (Cat. no.: 3-000-203-G/X; Drummond, USA) and Nanoject II or Nanoject III (Cat. No.: 3-000-207 and 3-000-204 respectively; Drummond, USA). Stereotaxic coordinates relative to bregma for specifically targeting the EVN were as follows: anterior/posterior -5.80mm; lateral +0.60mm; depth from brain surface -4.4 and -4.3mm. Each depth received 100nl of AAVs or 200nl of rabies (CVSN2c-ΔG-EnvA-mCherry, Sainsbury Wellcome Centre Virology Core Facility) depending on the surgery. Following viral injection, the skin was closed with sutures (Cat. No.: VR493; Ethicon, USA).

Subsequent rabies injection or histology began no sooner than 14 days after the initial injection of AAVs to allow sufficient time for their expression. Likewise, histology following rabies injections also took place 14 days after rabies injection for the same reason.

#### Retrograde labelling

To identify EVN neurons fluorogold was injected into the right horizontal semi-circular canal of ChAT-Cre::tdTomato or Chat-Cre mice. These retrograde injections were made as described previously (Mathews et al., 2015). Briefly, mice were anaesthetised with 4% isoflurane in oxygen, given appropriate analgesics and maintained on 2% isoflurane in oxygen during surgery. The area behind the ear was shaved and cleaned, and an incision was made 1mm behind the right pinna. The muscles were separated such that the horizontal semi-circular canal was visualised. A 23G needle was used to thin the bone of the horizontal semi-circular canal until a small hole (approximately 100μm) was made. A 29G needle attached to a 1ml insulin syringe was used to carefully introduce 100nl of 2% fluorogold (Sigma-Aldrich) in saline into horizontal canal and the skin sutured. Animals were sacrificed 3 days later by transcardial perfusion described below.

### Tissue preparation and histology

Mice were deeply anaesthetised with intra-peritoneal injection of pentobarbital (200mg/ml concentration at 10mg/kg dosage; Dolethal) and subsequently transcardially perfused with 0.1M phosphate buffer saline (PBS, pH 7.4) followed by 4% paraformaldehyde in 0.1M PBS. The brains were harvested and immersion-fixed in 4% paraformaldehyde in 0.1M PBS for a minimum of 2hr at room temperature. 50μm thick coronal brain sections were sliced on a Leica V1200 vibratome and collected and stored in tissue culture wells filled with 0.1M PBS and 0.01% sodium azide solution until use.

Free-floating sections were incubated in primary antibodies diluted in 0.1M PBT (0.1M PBS with 0.1% Triton X-100 and 1% BSA) overnight. They were then washed three time in 0.1M PBS for 10 mins each before secondary antibody incubation (diluted in 0.1M PBS) for 2hrs. After washing again three times with 0.1M PBS for 10mins each, the sections were mounted onto Superfrost+ (Thermo Scientific) glass slides, dried and cover-slipped with DAPI-Fluoromount (SouthernBiotech 0100-20). Primary and secondary antibodies used for different AAV and rabies surgeries are summarised in Table 1. All sections were imaged using the Zeiss AxioScan Z1 slide scanner or Zeiss AxioImager at 10x or 20x magnification.

### Cell counting

#### Manual

For monosynaptic rabies tracing, the cell numbers of ‘starter’ EVN neurons (defined as expressing both the nuclear GFP from the AAV and mCherry from the RABV) was determined by eye, focusing through 50 μm thick coronal sections containing the EVN and counting the nucleoli identified with DAPI staining in consecutive sections containing the EVN.

#### QUINT workflow

In rabies tracing experiments where manual cell counting was not practical, cells were quantified and spatially analysed in a semi-automated manner using open-source software in QUINT workflow (detailed in Yates et al., 2019). Briefly, serial sections were registered to a 3D reference atlas (Allen Brain Mouse Atlas 2017) using QuickNII software. These sections are then segmented into cell, tissue and background using ilastik software. Finally, Nutil software was used to merge custom atlas maps from QuickNII with segmented images from ilastik to quantify cells and their coordinates. These data were exported to Microsoft Excel for further analysis. All output cells and their locations were cross-checked against original JPEG images of sections to ensure accurate counting and location reporting – here, mislabelling was manually adjusted. Importantly, the Allen Brain Mouse Atlas 2017 used in QuickNII does not include the location for the EVN and so EVN cells were misplaced as medial vestibular nucleus neurons. These were manually adjusted in post-processing.

### Behaviour recording and analysis

Behavioural assessment of TeLC expressing and control mice behavioural recordings took place at 7, 10 and 14 days post-AAV surgeries (see vectors listed above for details).

#### Open field

Mice were allowed to freely explore a square white Perspex arena for a period of 5 minutes while being recorded by an overhead Doric Behaviour Tracking video camera recording at 30 frames per second. To avoid effects of habituation on experiments mice were exposed to the arena for 5 minutes on the three days prior to experimental recordings.

Videos were analysed in EthoVision XT (Noldus) video tracking software. Here, centre-point tracking feature was used to track and generate total distance travelled as well as heat maps of movement where pixel colour represents total time spent at the location (more time spent corresponds to lighter blue colour). Distance travel data was exported to Microsoft Excel for plotting and further analysis. TeLC and control conditions were compared using student’s paired T-test.

#### Balance beam

The balance beam test (adapted from Tung et al., 2014) was used to measure subtle motor changes in mice where EVN transmission was blocked. The beam (1cm square; 83cm in length) was elevated and suspended between two platforms of different heights (13cm on the left and 19cm on the right) to create a slight incline of 3 degrees. On the right-hand side, the platform included a stage for mice to walk to and rest. A mirror was placed below and angled so to reflect the underside of the beam and mice onto the video recorder. Mice were habituated and trained to walk on the beam prior to AAV injections by placing the mouse progressively further away from the end platform and stage. Video recording from the right-side view of the mouse was carried out with a high-speed camera (Ximea) recording at 200 frames per second.

Video were analysed in MaxTraq Software (Innovision Systems) for speed of traversing across the beam and the nose to tail angle of each mouse measured at the centre of the beam. Tracking data was exported to Microsoft Excel for plotting and further analysis. TeLC and control conditions were compared using student’s paired t-test.

## Results

### Molecular targeting of EVN neurons

EVN neurons are known to co-localise ChAT and CGRP in mice (Ohno et al., 1991; Mathews et al., 2015). We used this information to design an intersectional genetic strategy to selectively target these neurons via a stereotaxic injection into the brainstem. First, we anatomically visualised EVN neurons, which are known to project and synapse on to vestibular hair cell receptors and primary afferents in the peripheral sensory apparatus, by injecting the retrograde tracer fluorogold into the horizontal semi-circular canal of ChAT-cre::tdTomato mice.

**Figure 1:**
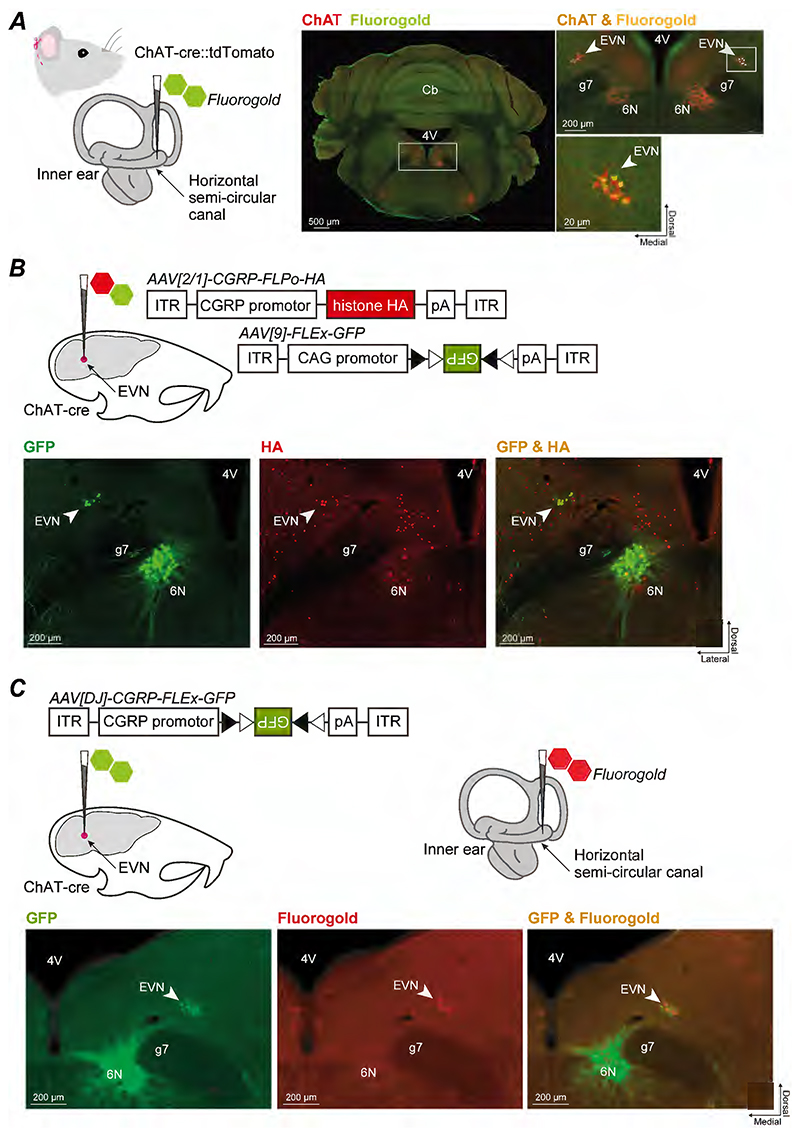
Molecular-genetic targeting of EVN neurons. ***(A)*** The fluorescent retrograde tracer fluorogold (FG) was injected into the horizontal semi-circular canal of ChAT-cre::tdTomato mice. Gold FG staining overlaps with red ChAT-positive, putative EVN neurons in red (arrow, insets), confirming EVN is located dorsolateral to the genu of the seventh cranial nerve (g7), and can be targeted with ChAT-cre animals. ***(B)*** In ChAT-cre mice, a 1:1 ratio of cre-dependent AAV expressing GFP and a HA-expressing AAV (red) under control of the CGRP promoter was stereotaxically injected into the EVN. Yellow overlap (arrow, far right image) confirms that CGRP and ChAT, can be used to selectively target EVN neurons. ***(C)***. Combined strategy where a single AAV combines the CGRP promoter and a cre-dependent FLEX switch can be used to target EVN neurons, confirmed by stereotaxic injection of this AAV into the EVN and fluorogold injection into the semicircular canal. GFP and fluorogold co-localisation observed on average (± standard deviation) in 15±4 neurons (n=5 animals), representing approximately 40% of the EVN. *Abbreviations*: *4V* fourth ventricle; *Abbreviations*: *4V* fourth ventricle; *6N* sixth cranial nucleus (abducens); *CAG* chicken beta-actin; *Cb* cerebellum; *CGRP* calcitonin gene related peptide; *ChAT* choline acetyltransferase; *EVN* efferent vestibular nucleus; *ITR* inverted terminal repeats; *pA* poly A. Mouse inner ear schematic adapted from Schutz *et al*. (2014).

Fluorogold labelling of EVN neurons, which are located dorsolateral to the genu of the seventh cranial nerve, was observed overlapping with ChAT-positive putative EVN neurons, confirming that EVN neurons can be targeted using cholinergic labelling in ChAT-Cre animals (Figure 1A). However, cholinergic neurons are also observed in the nearby abducens nucleus limiting the ability to target the EVN with stereotaxic injections alone. We therefore attempted to further refine this targeting by complementing the cre line with an AAV that only expressed a transgene in neurons that were both ChAT (cre) and CGRP positive. This AAV used a CGRP promoter fragment to express a nuclear tagged human influenza hemagglutinin (HA) in CGRP-positive cells. We used this in combination with a Cre-dependent AAV to express GFP in ChAT-positive cells in ChAT-Cre mice. Stereotaxic co-injections of both AAVs in ChAT-Cre mice allowed us to visualise ChAT-positive cells in green and CGRP-positive cells in red. We observed co-localisation of GFP and HA in EVN neurons (Figure 1B).

Next, we developed a cre-dependent AAV using this CGRP promoter to selectively express GFP in cells that are both ChAT and CGRP positive. Stereotaxic injections of this AAV allowed us to visualise GFP in EVN neurons. To ensure the AAV effectively targeted EVN neurons, we performed fluorogold injections in the horizontal semi-circular canal. Co-localisation of GFP with fluorogold was observed on average (± standard deviation) in 15±4 neurons (n=5 animals), representing approximately 40% of the nucleus, confirming this strategy selectively targets EVN neuron (Figure 1C).

### Monosynaptic inputs to EVN neurons

**Figure 2:**
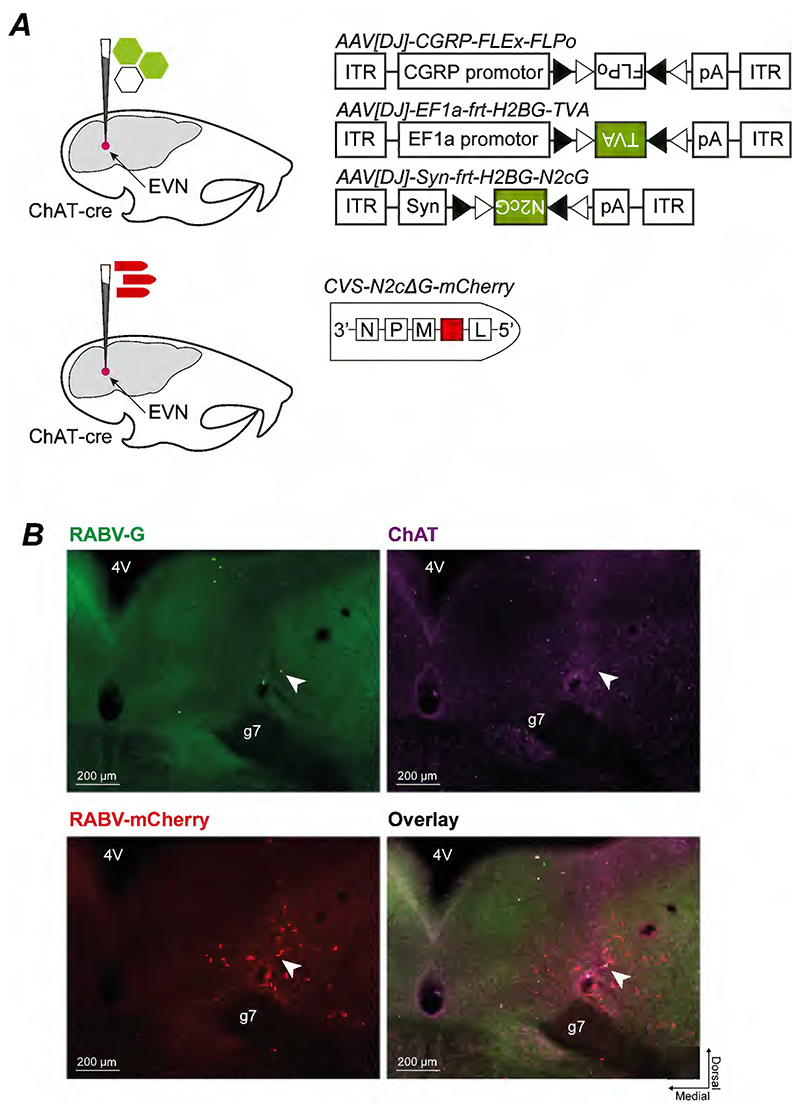
Monosynaptic rabies tracing of EVN neuronal inputs. ***(A)*** Strategy for rabies tracing in ChAT-cre mice where first avian receptor TVA (AAV[DJ]-EF1a-frt-H2BG-TVA) and rabies N2c glycoprotein (AAV[DJ]-Syn-frt-H2BG-N2cG) were stereotaxically injected with AAV[DJ]-CGRP-FLEx-FLPo in a 1:1:1 ratio to prime neurons for rabies tracing. Two weeks following initial rAAV injections, EnvA-psudotyped, glycoprotein-deficient rabies tagged with red fluorescent protein (EnvA-CVS-N2cΔG-mCherry) was stereotaxically injected to the same coordinates. ***(B)*** Successful transduction (arrow, overlay) of rabies glycoprotein (arrow, RABV-G), and rabies virus (arrow, RABV-mCherry) in a ChAT-positive EVN neuron (ChAT, arrow). A total of 5 starter EVN neurons were identified across 3 mice. *Abbreviations*: *4V* fourth ventricle; *EVN* efferent vestibular nucleus; *g7* seventh cranial nerve.

Selective expression of recombinases, such as flp recombinase, in genetically defined neurons provides a means to interrogate both their anatomy and function (Nectow and Nestler, 2020). Our intersectional approach provided a means to selectively target EVN neurons, and therefore we developed a viral vector (pAAV-CGRP-FLEx-FLPo) that combined the short CGRP-promoter and cre-conditional expression to drive flp-recombinase only in EVN neurons (Figure 2A). We first used this tool to identify direct synaptic inputs to EVN neurons.

We co-injected a mixture of three AAVs into ChAT::Cre mice – AAV[DJ]-CGRP-FLEx-FLPo to drive selective FLPo expression only in EVN neurons, and AAV[DJ]-Ef1a-frt-H2BG-TVA and AAV[DJ]-Syn-frt-H2BG-N2cG to express the TVA receptor and rabies glycoprotein respectively in a Flp-recombinase dependent manner. The TVA receptor permits selective cell entry of EnvA-pseudotyped rabies virus, while rabies glycoprotein expression enables monosynaptic transfer of rabies virus (Reardon et al., 2016). 2 weeks following this AAV injection into the region of the EVN, we injected RABV-N2c-EnvA-mCherry at the same stereotaxic coordinates to target EVN neurons and identify their monosynaptic inputs (Figure 2B).

**Figure 3:**
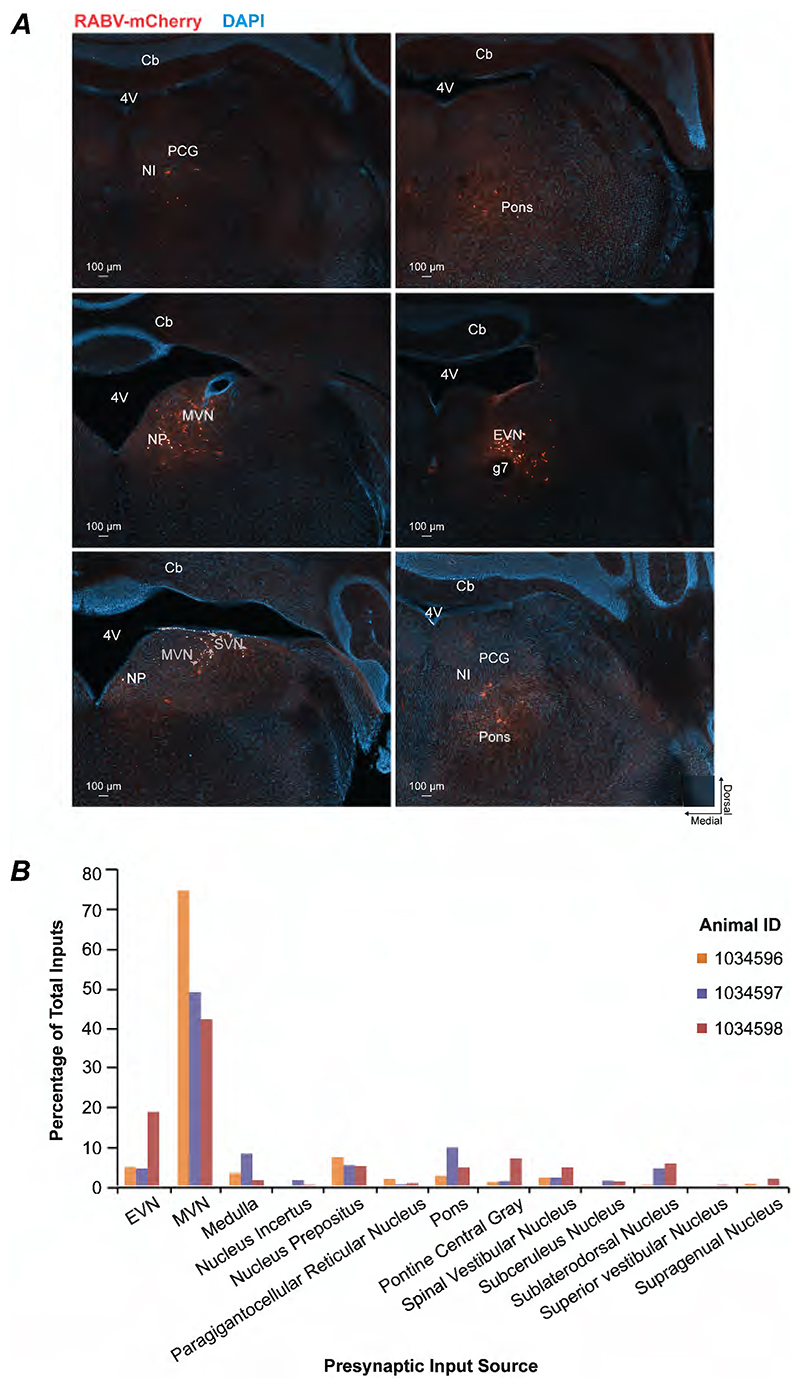
Identity and relative contribution of monosynaptic input sources to EVN starter neurons. ***(A)*** Example input regions from rabies tracing shown in red. ***(B)*** The 13 conserved input sources and their relatively weighting across animals (n=3) form the majority (62%) of all inputs observed. Comparing animals, Pearson correlations were high (r = 0.91–0.97) and Spearman correlations were moderate to high (r = 0.59–0.71), reflecting alignment across proportional weighting and conserved ranking of dominant inputs respectively. *Abbreviations*: *4V* fourth ventricle; *Cb* cerebellum; *EVN* efferent vestibular nucleus; *g7* seventh cranial nerve; *MVN* medial vestibular nucleus; *NI* nucleus incertus; *NP* nucleus prepositus; *PCG* pontine central gray; *SVN* superior vestibular nucleus.

We identified five starter EVN neurons across three mice. These starter neurons were abundantly innervated from input regions (starter to input neuron ratio of 1:356). Almost all monosynaptic inputs to EVN neurons were found within the brainstem/midbrain region (such as vestibular nuclei, reticular formation, medullary and pontine regions and autonomic /arousal nuclei), suggesting modulation by local circuits (Figure 3A). No neurons were observed in cortical or thalamic regions, or in the spinal cord (data not shown). Details of all input sources and distribution are presented in Supplementary Tables 1 and 2.

The small number of EVN starter neurons provides the opportunity to assess the heterogeneity of inputs to different EVN cells. To assess the consistency of presynaptic inputs to the EVN, we compared the identity and relative contribution of input sources across animals (Figure 3B), and applied statistics on all animal pairs (1034596 vs 1034597, 1034596 vs 1034598, 1034597 vs 1034598). We observed 21 unique input sources, 13 (62%) of which were shared across the three mice (Jaccard index paired scores 0.65-0.79). This suggests a core network of inputs present: the medial vestibular nucleus (MVN), EVN, nucleus prepositus, pons, medulla, spinal vestibular nucleus, pontine central gray, sublaterodorsal nucleus, supragenual nucleus, subceruleus nucleus, paragigantocellular reticular nucleus, and superior vestibular nucleus. The MVN was the dominant input source, accounting for approximately 42–75% (normalised) of total presynaptic neurons across animals. Other shared sources contributed smaller but consistent fractions of total input. Moreover, Pearson correlations were high across all animal pairs (r = 0.91–0.97), indicating strong alignment across animals in the proportional weighting of inputs. Spearman correlations were moderate to high (r = 0.59–0.71), reflecting conserved ranking of dominant inputs with greater variability among lower-contributing nuclei. Together, these analyses demonstrate that EVN neurons receive input from a conserved set of presynaptic sources with highly similar distributions across animals. Variability across animals primarily reflects differences in the relative contribution of minor inputs rather than changes in the core afferent architecture.

### EVN inhibition in behaviour

**Figure 4:**
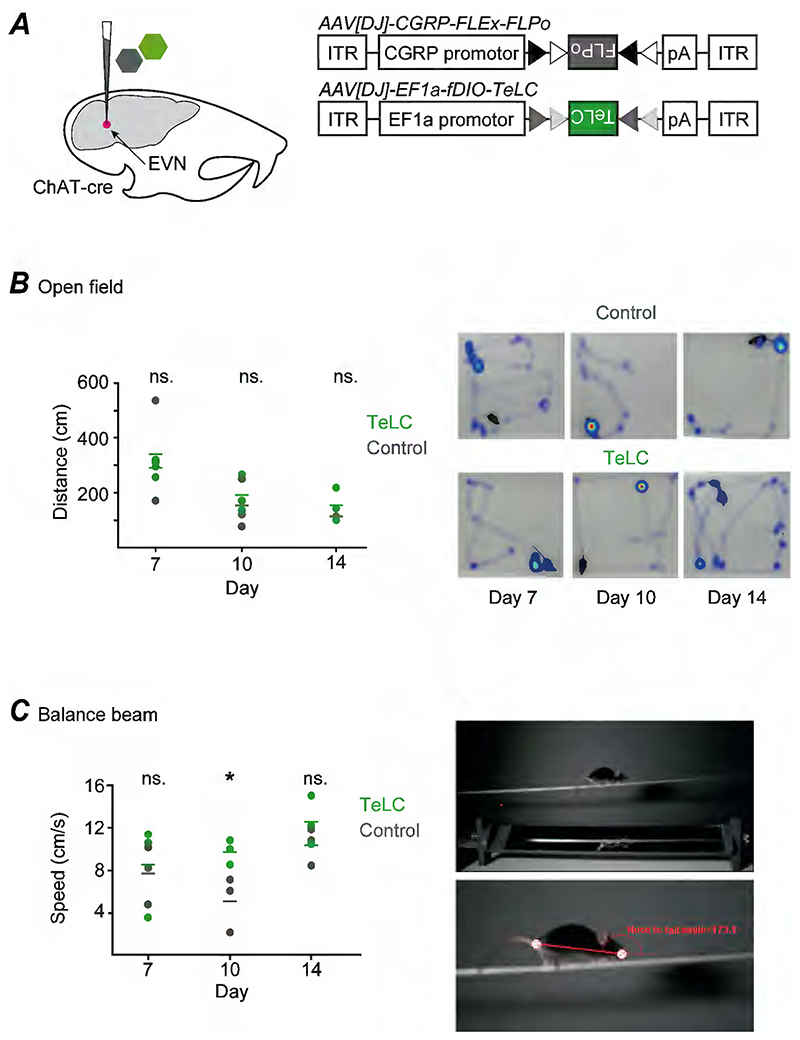
Selective disruption of EVN neurotransmission and behavioural assays. ***(A)*** Schematic of the strategy for selective block of EVN neurons. Tetanus toxic light chain (TeLC) was selectively expressed in EVN neurons to block their transmission in ChAT-Cre mice via stereotaxic co-injection of AAV[DJ]-CGRP-FLEx-FLPo and AAV[DJ]-EF1a-fDIO-TeLC-GFP in a 1:1 ratio (n=3). In control mice, TeLC was replaced with TeLC with AAV[DJ]-EF1a-fDIO-GCaMP6s (n=3). ***(B)*** No significant difference was observed across control and TeLC expressing mice in an open field (white Perspex box) arena in the first 60 seconds of recordings, measured on days 7, 10 and 14 following stereotaxic surgery. ***(C)*** TeLC expressing mice demonstrated a significantly faster time to traverse the balance beam than controls on day 10 following stereotaxic surgery in line with peak TeLC activity and exposure. *p<0.05.

A complete understanding of the function of any neural circuit requires the ability to selectively alter the synaptic output of that group of neurons. To demonstrate whether our novel method could potentially be used to block synaptic output from EVN neurons we combined the selective flp-recombinase expression described above with an AAV that drives the expression of tetanus toxin light chain (TeLC) in a flp-dependent manner (Figure 4A).

TeLC is a potent means to block synaptic transmission and does so via the cleavage of the synaptic protein VAMP2, preventing vesicle docking. AAV mediated expression of TeLC is known to completely block release of neurotransmitters with effects beginning ~10 days after AAV injection (Murray et al., 2011).

In experimental animals we performed bilateral co-injections of our EVN targeting AAV (AAV[DJ]-CGRP-FLEx-FLPo) with the Flp-dependent AAV[DJ]-EF1a-fDIO-TeLC-GFP into ChAT-Cre mice to selectively block synaptic transmission only from EVN neurons. Control animals received an injection of AAV[DJ]-EF1a-fDIO-GCaMP6s instead of TeLC at the same coordinates.

To assess whether our technique could be used to interrogate the behavioural role of EVN neurons, we performed two preliminary behavioural assays. Prior to formal behavioural assessment we examined animals for any gross behavioural abnormalities, especially those associated with peripheral vestibular abnormalities (Hardisty-Hughes et al., 2010). This included tilting of the head and circling. We also examined mice for other phenotypes such as reduced grooming and weight loss, but we did not observe any changes in these metrics (data not shown).

For a preliminary assessment of motor abnormalities in animals with disrupted EVN function we used an open field test. Mice were tracked in an open field arena (n=3 per condition) for 5 minutes and their path length, movement velocity and turning velocity analysed at 7-, 10- and 14-days post injection. No difference was found between control animals and those lacking EVN output (Figure 4B), suggesting the EVN is not required for gross motor function.

For a behavioural assessment that would require more engagement of the vestibular system we used the balance beam test, where animals with a vestibular impairment show balance deficits (Tung et al., 2014). As above animals were tested 7-, 10- and 14-days post AAV injection. Both control and EVN-disrupted animals were able to traverse the balance beam following a training period. No difference was found between groups in terms of the number of foot slips made by each animal as they walked across the beam, with both groups of animals making minimal slips per trial (foot slips per trial – day 7: control 0.4±0.4, TeLC 0.5±0.2; day 10: control 1±0.4, TeLC 0± 0; day 14: control 0.5±0.2, TeLC 0.17±0.17).

Animals with a disrupted EVN were consistently faster at traversing the balance beam (Figure 4C) (speed (cm/s) – day 7: control 6.8±1.1, TeLC 8.8±1.3; day 10: control 6.0±1.3, TeLC 10.4±0.9; day 14: control 8.6±1.0, TeLC 11.8±1.7). This reached statistical significance at 10 days following injection (Student’s t-test p=0.021). No other differences were found between control and EVN disrupted groups.

## Discussion

It is well accepted that efferent vestibular nucleus (EVN) neurons contribute to fine-tuning of vestibular sensory input by modulating firing patterns, signal timing and sensitivity of vestibular afferents and hair cells (reviewed in Mathews et al., 2020). However, the behavioural role of the EVS continues to be debated. An inability to target the EVN selectively has meant that probing its function in awake behaving animals has not been achievable. Here, we add to the toolkit for studying efferent vestibular system (EVS) function with a novel AAV-based EVN-targeting virus that draws on recent technological advances in neuroscience.

We combined a cre-recombinase driver line (ChAT::Cre) with a short AAV promoter active in CGRP-positive neurons to drive selective gene expression only in neurons which express both these genes. As EVN neurons are one of the few neuronal subtypes to co-express these genes in the brainstem, stereotaxic injection permitted their selective targeting. We used this intersectional strategy to express a separate recombinase, FLPo, selectively in EVN neurons. Paired with recombinase-dependent AAVs, this allows for the selective anatomical mapping and activity manipulation of EVN neurons. We used this to assess direct (monosynaptic) EVN inputs, and perform preliminary behavioural assessment of EVS function. Though our anatomical and behavioural studies restricted genetic interventions to modified rabies tracing and expression of TeLC, this same system can be used for other systems neuroscience interventions such as the selective expression of fluorescent proteins, or optogenetic or chemogenetic modulators (Nectow and Nestler, 2020) for further direct interrogation of EVS function.

### EVN-specific monosynaptic rabies tracing strategy

To date, this work represents the first monosynaptic tracing strategy selective to the EVN. We identified a small number of starter neurons (1–2 per animal; n = 3 animals). This reflects the technical challenges of targeting a very small brain region, the requirement for two stereotaxic surgeries, and the need for successful transduction by four viral vectors for a starter cell. As the precise mechanisms governing rabies virus transsynaptic transfer remain incompletely understood (Rogers and Beier, 2021), and because low starter cell numbers can disproportionately influence estimated input fractions (Tran-Van-Minh et al., 2023), our dataset may provide only a partial representation of EVN inputs. Nevertheless, the strong alignment of input strengths (Pearson correlation 0.91-0.97 across animal pairs) and conserved ranking of input regions (Spearman correlation 0.59-0.71 across animal pairs) of our data suggests that these regions constitute a core set of EVN inputs, though there are likely additional inputs not captured by the present approach.

It is also worth noting that previously, pseudorabies virus was used to assess polysynaptic inputs to EVN neurons in gerbils (Metts et al., 2006). The number of putative starter EVN neurons reported (less than 6) and the distribution of inputs at early time points aligns with our data, with strongest input from the MVN and reticular formation.

### Direct presynaptic inputs indicate EVS function as multimodal processing center

Mapping circuit architecture to function has become an important means to elucidate nervous system function. We know the EVN is sensitive to a heterogeneous input profile (Mathews et al., 2015) from a variety of brain regions (Zhou et al., 2018, Metts et al., 2006), and decades of research have either demonstrated directly or hypothesised EVN activation across a wide range of behavioural and physiological conditions. By quantitatively mapping the monosynaptic inputs to EVN neurons and integrating the known functions of these source regions, we infer that the EVN supports two distinct yet overlapping functional paradigms in vestibular efferent literature. The majority of total EVN inputs observed support (i) vestibular plasticity and gaze stabilization (~65%) while (ii) state-dependent gating (~16%) including predictive motor suppression (~13%) represent smaller but important functions (Supplementary Table 2).

We propose that the EVN is an important component of reflexive, adaptable vestibular sensorimotor processing. Specifically, the EVN is a context-sensitive, integrative processor of vestibular, motor and internal state information for dynamically calibrating vestibular signalling in real time. This hypothesis is consistent with the self-input we observed- the EVN likely uses an internal feedback system as an auto-tune mechanism when filtering diverse information, and accounts for the long-standing variability in EVN-linked behaviours. Further work describing the nature of these inputs (for example, neurotransmitter phenotype) would provide more colour on the context for EVN activation.

#### i Vestibular plasticity and gaze control

In addition to the MVN, the EVN receives input from oculomotor-associated nuclei, notably the abducens nucleus and nucleus prepositus, which are integral to horizontal gaze control and gaze holding, respectively. Collectively these regions integrate vestibular signals with motor commands for gaze stabilisation and in refining vestibulo-ocular reflex (VOR) gain and adaptation (Dietrich et al., 2017; McFarland and Fuchs, 1992; Mettens et al., 1994; Müri et al., 1996; Lisberger and Miles 1980). Their strong input indicates the EVN aligns peripheral vestibular encoding with centrally calibrated gaze shifts. In agreement with our anatomical data, early hypothesis of EVS function included anticipation of volitional head movement and the ensuing gaze shift (Goldberg and Fernàndez, 1980; Highstein, 1991; Brichta and Goldberg, 2000). Furthermore, the EVS has been suggested to have a role in vestibular plasticity, particularly regarding the vestibuloocular reflex (VOR) through signalling via alpha-9 nicotinic acetylcholine receptors (α9 nAChRs) expressed at efferent vestibular synapses on hair cells, which can elicit inhibitory responses in afferents (Elgoyhen et al., 1994; Hiel et al., 1996; Anderson et al., 1997; Holt et al., 2017; Zhou et al., 2013). This plasticity could be driven by high volume of MVN input observed in our tracing data as electrical stimulation and lesioning of MVN neurons in rats resulted in increased expression of ChAT positive EVN neurons (Zhou et al., 2018). Alternatively, CGRP could underlie EVN activity in the VOR. CGRP knock-out mice was associated with a significant reduction in the VOR sensitivity without any changes in the number of ChAT positive EVN neurons (Luebke et al., 2014). Further, Chang et al. (2021) reported unimpaired VOR function in α9 nAChRs knock-out mice suggesting an alternative mechanism. Future work investigating the properties of MVN inputs to the EVN would help elucidate the role of the EVN in peripheral vestibular plasticity.

Separately, the EVS has also been implicated in motion sickness symptoms. CGRP expression levels increased in the EVN in rats with motion sickness (Xiaocheng et al., 2012), α9 nAChRs knockout mice with an attenuated EVN showed reduced motion sickness symptoms (Tu et al., 2017), and young adult mice displayed EVN activation (measured via cFos) during motion sickness symptoms (Lorincz et al., 2023). It is broadly accepted that motion sickness symptoms arise out of sensory mismatch and conflict between visual, proprioceptive and vestibular information. EVN involvement is consistent with this model, particularly given its dense and selective innervation from brainstem and midbrain regions that integrate multimodal sensory information.

#### ii State-dependent gating

Inputs from brainstem regions involved in arousal, visceral regulation, and motor control suggests that the EVN plays a key role in state-dependent sensory gating of vestibular input. Inputs from arousal- and sleep-related nuclei (e.g., sublaterodorsal, subceruleus, and nucleus incertus) imply that EVN activity may be modulated across sleep-wake cycles or during shifts in attentional state, potentially allowing the vestibular system to prioritize or suppress sensory input as needed (Boissard et al., 2003; Fraigne et al., 2015; Ryan et al., 2011; Valencia Garcia et al., 2017). Autonomic and visceral centers, including the nucleus of the solitary tract and medullary inputs, could provide interoceptive context enabling the EVN to modulate vestibular sensitivity during challenges like nausea, hypotension, or cardiovascular changes (Andresen and Paton, 2011; Zoccal et al., 2014). Meanwhile, inputs from sensorimotor integration regions (pontine gray and supragenual nuclei) and postural control centers (spinal and lateral vestibular nuclei) support the idea that the EVN dynamically calibrates vestibular afferent output based on ongoing motor activity, body and head orientation, and valence (Biazoli et al., 2006; Xiao et al., 2023). Previous studies have shown EVN activity increases during aroused states in toadfish (Highstein and Baker, 1985), and vestibular reflex sensitivity is enhanced under threatening postural conditions in humans (Lim et al., 2016; Naranjo et al., 2016). Multimodal stimulation such as light touch, sound, visual stimuli effectively increase EVN discharge (Highstein and Baker, 1985; Highstein, 1991; Münz and Claas, 1991; Roberts and Russell, 1972).

There is also a considerable body of literature that highlights the role of the EVN in predictive motor gating of vestibular input. In line with this, we show input from vestibulospinal and reticulospinal systems including the pontine reticular formation and lateral vestibular nucleus (which receives both motor input directly as well as through the cerebellum; Witts and Murray, 2019). These areas are central to generating motor programs for locomotion, posture, and axial control (Takakusaki et al., 2016). Their input to the EVN could provide the route for efference copy information about impending self-motion, enabling the EVN to adjust the gain of vestibular signals during volitional movement. In fact, cholinergic efferent inhibition of hair cells at the lateral line periphery is well reported in zebrafish during swimming. In larval Xenopus and zebrafish, the EVS has been shown to attenuate afferent hair cell discharge in phase with locomotor activity (Chagnaud et al., 2015; Lunsford et al., 2019; Odstrcil et al, 2023), and in a direction-specific manner (Pichler and Lagnado, 2020). In the lateral line, the EVN functions to suppress reafferent noise while ensure sensory hair cells remain receptive to external stimuli to ultimately maintain perceptual clarity during self-generated motion. In light of the relative distribution of inputs we observed and since mammals do not possess a lateral line, it is possible that the EVN evolved to process vestibular-based input (~65%) more than motor-based input (~13%).

### Behavioural assessment of EVS function

We performed a preliminary behavioural assessment of EVS function via the selective expression of TeLC in EVN neurons. TeLC provides a potent block of synaptic transmission approximately 10 days after AAV injection (Murray et al., 2011). Importantly, animals were overtly normal and showed no health problems following EVN disruption. Additionally, animals showed no gross motor defects in the open field and were able to traverse a balance beam. This latter result potentially indicates that the EVS is not required for all peripheral vestibular function as this task is heavily dependent on the vestibular system and mice with vestibulopathies show clear deficits in this behaviour (Tung et al., 2014; Tung et al., 2016).

Mice with a disrupted EVN did show a mild phenotype of traversing the balance beam faster than control animals. This counterintuitive result (as you may expect that animals with a suboptimal vestibular system would be slower at a balance task) may indicate an inability of animals to regulate their own locomotor speed during challenging conditions, perhaps because of disrupted motor efference copy information (Chagnaud et al., 2015; Lunsford et al., 2019; Odstrcil et al, 2023). This result also hints that the EVN normally acts to constrain movement speed, acting as a behavioural brake or stabilizer during dynamic motor tasks, possibly at the cost of long-term sensorimotor adaptability or precision. However, further behavioural assessment will be required to confirm this result.

One possible explanation for the limited behavioural effects observed could be the small number of cells ultimately affected by virally delivered manipulations. The EVN has few neurons and only transducing a proportion of the cells within it results in a small overall intervention. Our CGRP driven EVN targeting isolated on average fifteen EVN neurons, approximately one third of all EVN neurons, making it challenging for behavioural assays where a complete inhibition of the whole EVN would provide more clarity in results. Additionally, given the highly plastic nature of the vestibular system and its ability to compensate for perturbations (Gittis and du Lac, 2006) the use of a chronic manipulation such as TeLC could obscure EVS function if the system were to compensate for this lack of output.

### Limitations of our approach

The EVN is a very small brain nucleus containing only ~41 neurons per hemisphere in mice (Mathews et al., 2015). Our technical approach requires up to 4 viruses to transduce the same neuron, in a brain region that is only a few hundred microns across. The result of this is that a small number of EVN neurons are targeted in our experiments. As discussed above the small number of rabies starter neurons could provide only a partial picture of synaptic input. Similarly, the relatively small number of neurons targeted with TeLC could mean that the remaining, functional, EVN can compensate for the loss of synaptic output from these neurons. The result of which could be some behavioural roles of the EVN are missed. Future refinements of this method should be targeted towards increasing its efficiency to complement the selectivity described here.

### Conclusions and next steps

Here, we provide a novel means of targeting and accessing the EVN that we use for monosynaptic tracing of inputs and that could be applied to behavioural assessments of EVS function. Our input mapping experiments utilise rabies tracing methodology and provide valuable new information of the anatomical organization of the efferent vestibular system. For the first time, we present a map of the type of information that the EVN directly receives and processes. Combined with the existing body of EVS research, our findings highlight the dynamic, multimodal nature of the EVN, acting as a hub to integrate and update the vestibular periphery in real time with relevant internal and external information to support overall vestibular health.

Our molecular genetic strategy could also be used alongside bespoke behavioural tests to further demonstrate this filtering role or test alternative functional hypotheses. For example, head-fixed eye tracking for VOR performance or complex movement tasks such as narrower or curved beam experiments could be adapted to our technology. Our system is also compatible for use with acute circuit interventions such as chemo- or opto-genetics and calcium imaging which could provide a clearer understanding of EVS function.

EVS research continues, and the novel methodology presented here could enable specific targeting of the EVN for functional research and, ultimately, faster progress in understanding this somewhat mysterious nucleus.

## Supplementary Material

Supplementary Material

## Figures and Tables

**Table 1:** Antibodies used throughout for immunohistochemistry experiments. Dilutions and catalogue number provided.

Experiments	Primary Antibody (dilution, catalogue number)	Secondary Antibody (dilution, catalogue number)
Figure 1	Chicken anti-GFP (1:1000, AB13970)	Anti-Chicken 488 (1:1000, A11039)
	Rabbit anti-FG (1:200, AB153-I)	Anti-Rabbit 594 (1:1000, A21207)
Figure 2	Mouse anti-mCherry (1:1000, AB125096)	Anti-Mouse 594 (1:1000, A21203)
	Sheep anti-ChAT (1:250, AB18736)	Anti-Sheep 647 (1:1000, A21448)
	Chicken anti-GFP (1:1000, AB13970)	Anti-Chicken 488 (1:1000, A11039)
Figure 3	Rabbit anti-TeLC (1:1000, AB53829)	Anti-Rabbit 647 (1:1000, A21245)
	Chicken anti-GFP (1:1000, AB13970)	Anti-Chicken 488 (1:1000, A11039)
