# Supplementary material for: Selective genetic targeting of the mouse efferent vestibular nucleus identifies monosynaptic inputs and indicates function as multimodal integrator
Source: J Neurophysiol. Author manuscript; Available in PMC 2026 Mar 17. (PMC7618883; doi:10.1152/jn.00467.2025)
Supplement: Supplementary Material [file EMS212819-supplement-Supplementary_Material.docx]

**Supplementary Data**

Supplementary data is available at:

<https://osf.io/8wz2g/files/osfstorage/6983745ad7eb2639cf4c4f46>
